# Cinnamon-Nanoparticle-Loaded Macroalgal Nanocomposite Film for Antibacterial Food Packaging Applications

**DOI:** 10.3390/nano13030560

**Published:** 2023-01-30

**Authors:** Samsul Rizal, H. P. S. Abdul Khalil, Shazlina Abd Hamid, Esam Bashir Yahya, Ikramullah Ikramullah, Rudi Kurniawan, Che Mohamad Hazwan

**Affiliations:** 1 Mechanical Engineering Department, Universitas Syiah Kuala, Banda Aceh 23111, Indonesia; 2 Bioresource Technology Division, School of Industrial Technology, Universiti Sains Malaysia, Penang 11800, Malaysia; 3 Green Biopolymer, Coatings and Packaging Cluster, School of Industrial Technology, Universiti Sains Malaysia, Penang 11800, Malaysia; 4 Bioprocess Technology Division, School of Industrial Technology, Universiti Sains Malaysia, Penang 11800, Malaysia

**Keywords:** cinnamon nanoparticles, seaweed, biopolymer films, food packaging, antibacterial

## Abstract

In addition to environmental concerns, the presence of microorganisms in plastic food packaging can be hazardous to human health. In this work, cinnamon nanoparticles incorporated with red seaweed (*Kappaphycus alvarezii*) biopolymer films were fabricated using a solvent casting method. Cinnamon was used as a filler to enhance the properties of the films at different concentrations (1, 3, 5, and 7% *w*/*w*) by incorporating it into the matrix network. The physico-chemical, thermal, mechanical, and antimicrobial properties of the cinnamon biopolymer films were obtained using dynamic light scattering (DLS), scanning electron microscopy (SEM), transmission electron microscopy (TEM), Fourier transmission infrared spectroscopy (FT-IR), water contact angle (WCA) measurement, thermogravimetric analysis (TGA), mechanical testing, and antimicrobial testing, respectively. The results showed that the addition of cinnamon nanoparticles to the film improved the morphological, mechanical, thermal, wettability, and antibacterial properties of the nanocomposite films. The cinnamon particles were successfully reduced to nano-sized particles with an average diameter between 1 nm and 100 nm. The hydrophobicity of the film increased as the concentration of cinnamon nanoparticles incorporated into the seaweed matrix increased. The tensile and thermal properties of the cinnamon seaweed biopolymer film were significantly improved with the presence of cinnamon nanoparticles. The biopolymer films exhibited good inhibitory activity at 7% cinnamon nanoparticles against *Escherichia coli* (*E. coli*), *Staphylococcus aureus* (*S. aureus*), and Salmonella bacteria with inhibition zone diameters of 11.39, 10.27, and 12.46 mm, indicating the effective antimicrobial activity of the biopolymer film. The functional properties of the fabricated biopolymer film were enhanced with the addition of cinnamon nanoparticles.

## 1. Introduction

Plastic products have become indispensable to our daily lives [1]. They are frequently utilized in packaging and other applications. Plastic rubbish is plastic that has been discarded after its usefulness has expired [2]. The majority of this plastic waste ends up in landfills, beaches, rivers, and oceans, with only a small amount being recycled [3]. Plastics will never degrade and will remain in the environment for decades, producing environmental issues. As a result, limiting the usage of plastic will ease environmental problems. The creation of biodegradable materials to partially replace polymers derived from petroleum could present benefits for both manufacturers and consumers [4].

New materials and technologies such as bio-based polymers have been intensively investigated to meet the rising need for biodegradable and natural materials. Bioplastic or biopolymer products can be made from raw materials obtained from agricultural or marine sources [5,6]. Numerous studies have been conducted on the creation of bioplastics from diverse sources, including plants, microorganisms, and animals [7]. While research on new materials for various purposes is ongoing, some, such as polylactic acid and starch-based polymers, are currently commercially available on a worldwide scale [8]. Although there are several renewable sources from which these components can be produced, their limited biomass supply and cultivation or synthesis pose challenges.

The bio-nanocomposite-based film’s strength is still insufficient and inappropriate for food packaging. Food spoilage can result from food-borne bacteria, particularly those found in meal packaging [9]. Thus, the waste of disposable plastic that might cause environmental difficulties can be reduced by merging a biopolymer with nanoparticles to form a biopolymer film. In recent years, research has focused on the development of seaweed-based films for use in food packaging applications [10]. The barrier and mechanical properties of seaweed derivatives such as alginate and carrageenan have been investigated for their use in film fabrication [7]. Due to the fact that seaweed is predominantly composed of protein and non-starch polysaccharides, these molecules could be used to create films and packaging [8]. Plastic takes a long time to degrade, whereas seaweed films are biodegradable and beneficial to the environment [11]. Seaweed is one such resource that can serve as a source of raw materials for the manufacture of packaging materials [12]. Seaweed offers numerous advantages over terrestrial plant sources, including its low price and natural abundance. In addition, because it is a non-terrestrial plant material, there is no need to harm valuable land or compete with food production [13]. Additionally, seaweeds are considered to have fewer effects on the food chain and to avoid exposure to chemicals and fertilizers [14].

Cinnamon has recently been used as a preservative due to its antimicrobial properties [15,16]. It is also known for its high total phenol content and antioxidant activity [17]. Cinnamon is one of the most effective antibacterial spices, and its main component, cinnamaldehyde, has minimum inhibitory concentrations (MICs) in vitro ranging from 0.05 to 5 L/mL [18]. Cinnamon has been shown to inhibit the growth of molds, yeasts, and bacteria when consumed in food products [19]. As a result, they could be incorporated into packaging materials or used as edible coatings to extend food shelf-life [20]. Previous works have utilized cinnamon essential oil, cinnamon extracts, or conventional cinnamon particles as fillers in their films. Oyenkami et al. previously demonstrated that cinnamon oil extract from reinforced cellulose nanofiber biopolymer films successfully improved the functional properties of the film [21]. In this study, we used cinnamon nanoparticles as a filler to enhance the properties of seaweed-based biopolymer films particularly for food packaging. The physical, chemical, mechanical, and thermal properties of the fabricated films were investigated using dynamic light scattering (DLS), scanning electron microscopy (SEM), transmission electron microscopy (TEM), Fourier transmission infrared spectroscopy (FT-IR), water contact angle (WCA) measurement, thermogravimetric analysis (TGA), and mechanical testing for properties such as the tensile strength, Young’s modulus, and elongation at break, according to the standard method. Finally, the antimicrobial properties of seaweed-based biopolymer films were investigated using the agar diffusion method. Therefore, the main objective of this research is to develop new biodegradable packing materials containing cinnamon as a natural antimicrobial compound to improve the shelf life and quality of food products.

## 2. Materials and Methods

### 2.1. Materials

In this study, the red seaweed (*Kappaphycus Alvarezii*) was from Sabah. The cinnamon powder was purchased from the local supermarket (Lotus’s Malaysia). Analytical-grade glycerol, which acts as a plasticizer, was supplied by Systerm Chemical Sdn. Bhd.

### 2.2. Preparation of Cinnamon Nanoparticles

The cinnamon nanoparticles were created using a mechanical ball-milling process at room temperature with a ball-to-powder weight ratio of 10:1. The milling time and speed were set at 72 h and 160 r/min, respectively. The temperature of the drying oven for the cinnamon nanoparticles produced by the ball-milling process was kept at 45 °C to prevent moisture and agglomeration.

### 2.3. Preparation of Seaweed-Based Nanocomposite Film Incorporated with Cinnamon Nanoparticles

The seaweed-based nanocomposite film incorporated with cinnamon nanoparticles was prepared in a beaker using 10 g of seaweed, 500 mL of distilled water, and 5 g of glycerol. The nano-cinnamon powder was added to the mixture in various amounts (1%, 3%, 5%, and 7% wt%). The mixture was then placed in a blender and blended for 3 min to create a homogeneous seaweed slurry. The slurry mixture was then constantly stirred on a hot plate at a speed of 500 rpm and a temperature of 190 °C. The hot solution was allowed to cool for 5 min at room temperature before being poured onto a casting tray and dried for 24 h at 45 °C. The dried seaweed-based nanocomposite film incorporated with cinnamon nanoparticles was peeled off the tray and immediately desiccated at 50% RH for at least 48 h before further analysis and testing.

### 2.4. Characterization of Seaweed/Cinnamon Biopolymer Films

The particle size of cinnamon nanoparticles was measured using dynamic light scattering (DLS) on a Malvern Zetasizer Nano ZS Ver. 7.11 (Malvern Instruments, Malvern, UK). The detector and laser were aligned, and the background was calibrated for each measurement. The size distribution was quantified as the relative volume of particles in size bands represented by size distribution curves. In order to investigate the morphology of cinnamon nanoparticles, transmission electron microscopy (TEM) was performed using an instrument that was manufactured by Libra-Carl Zeiss and featured energy-filtered transmission electron microscopy. Before using TEM, the cinnamon nanoparticles were dried in an oven at 60 °C for 1 h. The preparation of the cinnamon nanoparticles was carried out in acetone, and they were then dispersed using an ultrasonicator for a period of 10 min. When preparing the samples for TEM examination, a drop of colloidal dispersion containing nanoparticles of cinnamon was deposited onto a carbon-coated copper grid. This step was followed by the preparation of the samples. Prior to being subjected to an examination by a TEM instrument under controlled conditions, embedded POA nanoparticles were allowed to air-dry at room temperature.

Fourier transform infrared spectroscopy (FT-IR) was used to analyze the functional groups and new bonds involved in the nanocomposite film. The FT-IR test was performed using the Shimadzu IR Prestige-21 machine. Cinnamon powder samples were prepared and oven-dried in the oven at 60 °C for 24 h before FT-IR analysis. The functional groups and new bonds in the nanocomposite film were examined using Fourier transform infrared spectroscopy (FT-IR). Before FT-IR analysis, film samples were cut into (3 × 3 cm) squares and oven-dried for 24 h at 60 °C.

The thermal stability of nanocomposite films was determined using a MettlerToledo thermogravimetric analyzer (TGA/DSC 1, Switzerland) between 30 °C and 800 °C at a heating rate of 20 °C/min in a nitrogen atmosphere. For each analysis, approximately 6 mg of the sample was placed in an alumina sample cup, and the empty cup served as a reference. The TGA curve yielded the sample weight loss (%) and char residue, whereas the DTG curve yielded the maximum decomposition temperature (T_max_).

The water contact angle was measured using the sessile drop method on a KSC CAM (KSV Instruments Ltd., Espoo, Finland) at room temperature. A syringe containing approximately 5 mL of water was injected onto the film surface. After the water was dropped onto the film surface, images were shot and documented immediately. To determine the average, two measurements were taken at different locations on the films with a sample size of 2.5 cm × 7.5 cm.

Using a tensile test (TS), the mechanical properties of nanocomposite films, including tensile strength (TS), Young’s modulus (YM), and elongation at break (EB), were measured at room temperature using a Texture Analyzer equipped with a 30 kg load cell in accordance with the ASTM D882-02 standard method. Using a utility knife, five 10 mm × 100 mm samples were cut from each nanocomposite film. The samples were initially conditioned in a desiccator at 23 °C and 50% RH prior to being tested in accordance with the standard. The initial grip separation and test velocity were established at 100 mm/s and 10.0 mm/s, respectively.

The fracture surface morphology of nanocomposite films was determined using scanning electron microscopy (Leo Supra 50 VP Field Emission, CarlZEISS SMT, Oberkochen, Germany). Before testing, film samples were prepared by drying them at 60 °C for one night. Before SEM analysis, film samples were adhered to the SEM pin holder with double-sided carbon adhesive tape. The film samples were then coated with a thin layer of gold prior to imaging to increase their electrical conductivity. The surface films were analyzed at 100–500× magnifications and 15 kV accelerated voltage.

Three food pathogens were used to test the antimicrobial activity. The pathogens chosen for the test were *E. coli*, *S. aureus*, and *Salmonella*. Bacterial samples were cultured for 24 h in sterile Mueller–Hinton broth at 37 °C. The test was performed on a punched film with a 6 mm disc diameter, and the antimicrobial activities were determined using the agar diffusion assay. Each bacterium was transferred to a Mueller–Hinton Agar (MHA) plate using a Finnpipette^TM^ F2 Variable Volume Pipette. L-Shaped Cell Spreaders were used to distribute samples on agar surfaces. Bacterial cultures were grown overnight on inoculated agar plates. They were then incubated at 37 °C for 24 h. The diameter of the inhibitory zone was measured with a caliper and expressed in millimeters.

## 3. Results and Discussion

**Figure 1:**
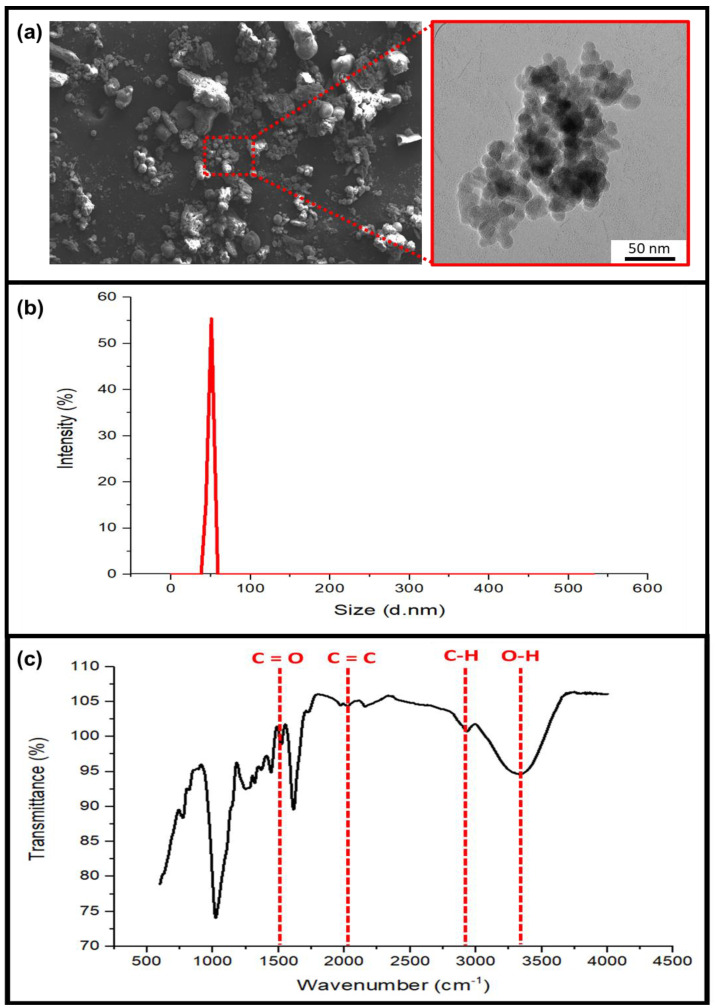
(**a**) SEM and TEM, (**b**) particle size distribution, and (**c**) FT-IR of cinnamon nanoparticles.

The cinnamon powder was converted into nanoparticles using ball milling. Figure 1a depicts typical SEM and TEM images of cinnamon particles. SEM and TEM were used to examine the sizes and shapes of the cinnamon particles. The results show that they were spherical and nano-sized. Figure 1b presents the particle size distribution range of cinnamon nanoparticles determined using a particle size analyzer. From the figure, it is observed that the nano-size distribution intensity of cinnamon particles reached around 90%, with an average cinnamon particle diameter ranging between 1 nm and 100 nm. These findings indicate that the majority of the cinnamon particle sizes had been reduced to the nanoscale due to the high intensity of the nano-size distribution [22]. As a result, both analyses indicate that the cinnamon particles were in the nano range.

The FT-IR spectra of nano cinnamon powder are observed in Figure 1c. Nano cinnamon powder’s primary absorption peaks were observed at the following wavelengths: 3329.14, 2931.80, 2158.35, 1612.49, 1517.98, 1442.75, 1369.46, 1317.38, 1249.87, 1022.27, and 773.46 cm^−1^. Peaks between 3400 and 3600 cm^−1^ are related to the hydroxyl groups’ vibrational O-H stretching [23]. This proved that the cinnamon was linked to a few molecules of water. There was another absorption band at 2931.80 cm^−1^ attributed to the asymmetric stretching vibration of C-H in methylene and methyl groups. The peak at 1573 cm^−1^ is assigned to the vibration of the aromatic ring C=C skeleton of an aromatic substance. The peak at 1450 cm^−1^ is very characteristic of an alcohol C–OH group within the bending vibration absorption [24]. A C=O aromatic structure represented by a transmittance peak at 1517.98 cm^−1^ was seen during stretching vibration, and the intensity could be connected to the stretching vibrations of C-H (methyl and methylene) at 1442.75 cm^−1^. The peak at 1727 cm−1 represents the carbonyl bond, and it is attributed mainly to the aldehydes of saturated fatty acid signatures in the sample [25]. Syringyl and guaiacyl units account for the vibration bands at 1249.87 cm^−1^ and 1022.27 cm^−1^. Later, a peak at approximately 773.46 cm^−1^ was attributed to the out-of-plane bending vibration of the aromatic C-H structure [26].

**Figure 2:**
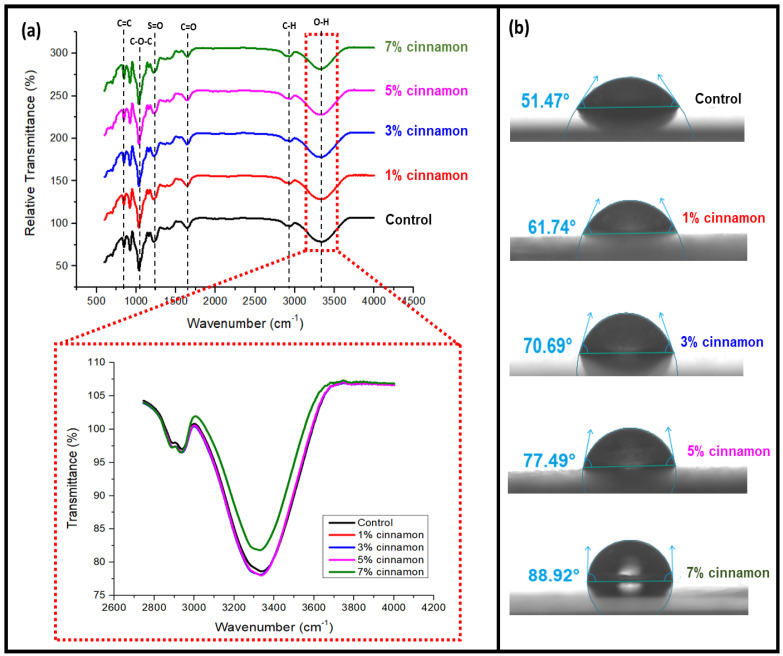
(**a**) FT-IR analysis of seaweed-based cinnamon film. (**b**) Contact angle thickness of seaweed-based film incorporated with cinnamon powder.

Figure 2a presents the infrared spectra of seaweed film incorporated with nano cinnamon and the control film. The large peak seen at roughly 3329 cm^−1^ was attributed to the presence of hydroxyl groups (O-H) in seaweed based on the FT-IR spectrum for the overall seaweed film. Cinnamon nanoparticles have a lower wavenumber compared to the control film, which reveals molecular changes due to the interaction between nano cinnamon and seaweed. The band measured at 2939 cm^−1^ was attributed to the lengthening of C-H [27]. Meanwhile, carbonyl group (C=O) stretching was indicated by a peak at about 1543 cm^−1^. Li et al. [24] associated the appearance of these peaks with cinnamaldehyde and other aldehydes found at high levels in cinnamon bark. The peak at 1573 cm−1 corresponds to the skeleton vibration of C=C of an aromatic ring, normally associated with eugenol, a phenol present in cinnamon oil. This is referred to as the stretching of carboxyl groups in the sulfated polysaccharides of seaweed. The sulfate ester-stretching of the kappa-carrageenan backbone is responsible for a peak at approximately 1217 cm^−1^, which was used to confirm the presence of carrageenan sulfated polysaccharide [28]. This group reflects macroalgae’s gelling characteristics. For raw seaweeds, a band that formed at roughly 1033 cm^−1^ revealed the glycosidic linkage in all carrageenan types. The peaks at about 921 cm^−1^ and 846 cm^−1^ were attributed to the presence of 3,6-anhydrous-D-galactose (DA) and D-galactose-4-sulfate (G4S), respectively [29]. The FT-IR spectra of biocomposite films used in this study include the distinctive bands of cinnamon nanoparticles and seaweed, as well as their interactions.

One of the common ways to determine how permeable a surface or material is to water is through the contact angle. Figure 2b shows the water contact angles (WCAs) of seaweed films incorporated with cinnamon nanoparticles. The results show that the fillers can enhance the WCA of the film. Similar behavior was observed in carrageenan films that also contained inorganic fillers, such as clay and silver nanoparticles (AgNPs), which were shown to improve the CA of neat films [29]. Additionally, a greater WCA value leads to improved solid surface hydrophobicity [2]. This may be explained by the fact that fillers used in the films have increased hydrophobicity, which enhances the hydrophobicity of seaweed-based films filled with fillers [29]. The composite films containing 7% cinnamon nanoparticles had the best contact angle value (88.92°) out of all the films, which minimized water interactions with the film surface and created a solid water droplet. This finding demonstrated that when the cinnamon nanoparticle loading increased, the interfacial adhesion of the nanofillers and the seaweed likely improved. As the concentration of cinnamon in the matrix component increased and the surface roughness decreased, the hydrophobicity of the films also increased [18]. Following the insertion of a 1% concentration of cinnamon nanoparticles with an equivalent contact angle of 61.74°, the film’s increased hydrophobicity was noticed. The surface roughness decreased when the content of cinnamon was raised, and the hydrophobicity of the film rose as well. This apparent trend reached its peak following the addition of 7% cinnamon particle nanofiller [18].

**Figure 3:**
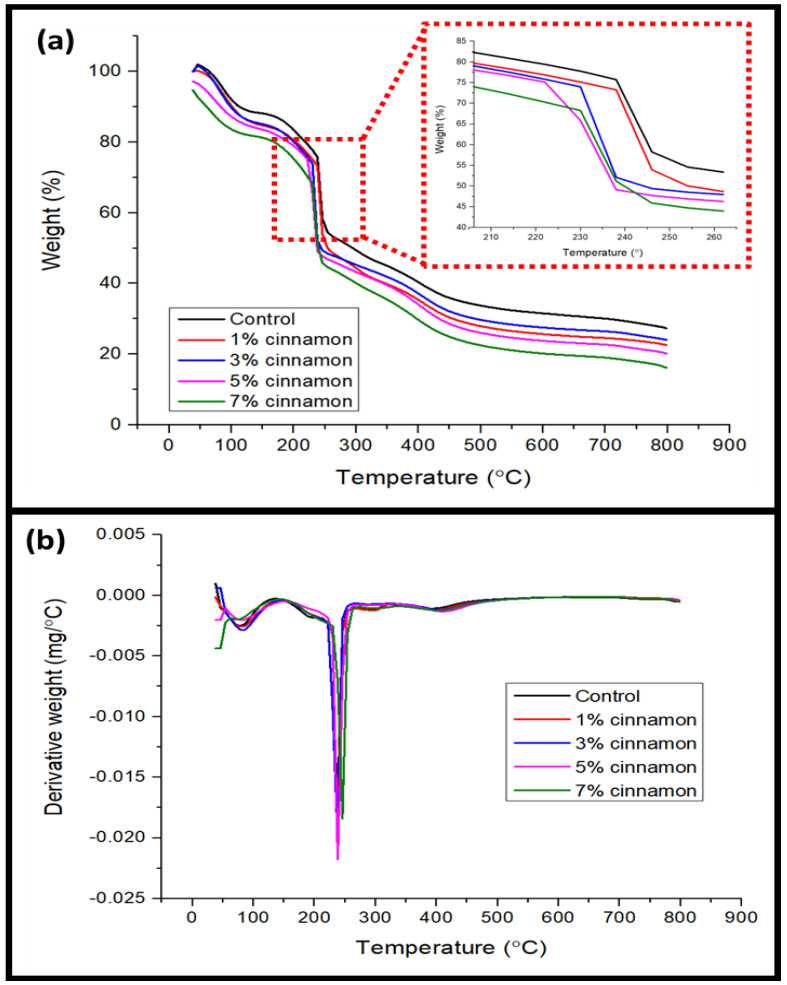
(**a**) TGA and (**b**) DTG curves of seaweed films incorporated with cinnamon nanoparticles.

The thermal degradation properties of seaweed films incorporated with cinnamon nanoparticles were analyzed using thermogravimetric (TG) analysis, as presented in Figure 3a. These seaweed films containing 7% cinnamon nanoparticles had a lower moisture content than the control film due to the equivalent weight losses. Furthermore, the highest volatilization of glycerol (a plasticizer) from the biopolymer matrix was attributed to the second stage of the thermal degradation of the films, which was seen in the temperature range of around 170 °C to 232 °C [30]. The primary step of film degradation, which was represented by the third stage in the TG feature, took place between 240 °C and 340 °C and is related to the thermal disintegration of the seaweed polymer. Finally, the highest deterioration of cinnamon nanoparticles at temperatures between 360 °C and 410 °C might be related to the fourth phase of thermal degradation. However, the glycerol removal from the film does not correspond to the thermal performance of the film.

**Figure 4:**
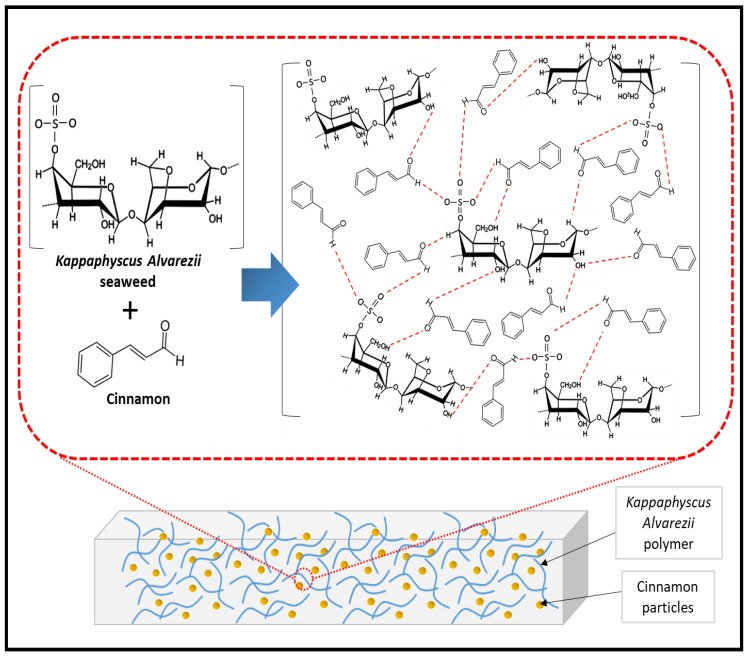
Possible mechanism (hydrogen bonding) of cinnamon incorporated with *Kappaphyscus Alvarezii* biopolymer.

The weight loss and derivative weight loss (DTG) as a function of the temperature of the cinnamon-nanoparticle-filled and neat marine algae films are shown in Figure 3b. As the concentration of cinnamon nanoparticles in seaweed films increased to 5% and declined thereafter, the starting temperature of decomposition (Ton) and the maximum temperature of decomposition (Tmax) were transferred to higher temperatures. The maximum rate of degradation occurred at a temperature of 222 °C, which was pushed from the onset temperature of 206 °C to an average of 230 °C. This demonstrated that the inclusion of cinnamon nanoparticles improved the thermal stability of the seaweed films up to the optimal concentration of the filler [31]. Better thermal stability of the material was indicated by a higher thermal breakdown temperature. Since Ton and Tmax were higher in films containing cinnamon nanoparticles than in those without, they had improved thermal stability. For films containing 5% cinnamon nanoparticles, the optimal Ton and Tmax were found at 225 °C and 230 °C, respectively. Stronger intermolecular interactions created by hydrogen bonding between the seaweed matrix and the cinnamon may be credited for this improvement, as displayed in Figure 4 [21]. This led to increased Ton and Tmax because it took more thermal energy to break the intermolecular bonds. Additionally, better lignin–matrix contact might be supported by the dispersion agent (glycerol), which would promote heat degradation.

**Figure 5:**
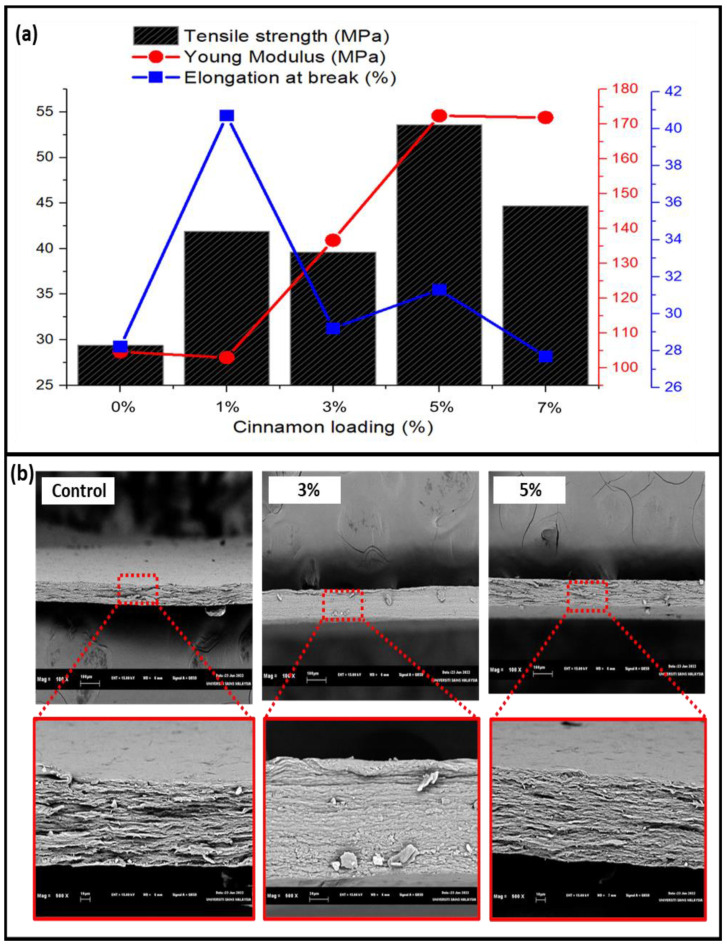
(**a**) Tensile properties of the fabricated seaweed-based cinnamon film, including tensile strength, Young’s modulus, and elongation at break. (**b**) Fracture surface morphology of the seaweed-based cinnamon particle film.

The nature of the substance in the solution and the thickness of the seaweed film both affect the tensile strength of the film. Because the seaweed film has a higher tensile strength, it can sustain pressure better and is less brittle. The strength to withstand pressure improves in films with a high tensile strength value [32]. The mechanical properties of seaweed film were investigated to determine the tensile strength (MPa) and elongation at break (%). As seen in Figure 5a, the film’s tensile strength increased as higher cinnamon nanoparticle concentrations were added. This might be because the film’s matrix elements interact more effectively. In this analysis, the results show that the 5% cinnamon nanoparticle sample had the maximum tensile strength, which was 53.606 MPa. The strong tensile strength of the material was matched by its low (31.294%) elongation at break value. This demonstrated that seaweed-based films with cinnamon nanoparticles as fillers could withstand greater loads or stresses than the seaweed film without the filler and had higher mechanical strengths, rigidities, and flexibilities. The composition of seaweed-based films, where carrageenan from red seaweed (*Kappaphycus alvarezii*) can help build a robust film with improved gelation capabilities, can be attributed to their higher strength. Additionally, it has been observed that an improvement in the filler’s mechanical qualities contributed to more acceptable interfacial adhesion between the matrix and fillers due to strong intermolecular contact, which was made possible by the hydroxyl groups in red seaweed and cinnamon nanoparticles [33]. Because of the fillers’ effective dispersion in the matrix, the enhanced mechanical characteristics also revealed that the cinnamon nanoparticles and red seaweed acquired favorable compatibility with one another. Glycerol as a plasticizer improves the matrix’s miscibility. The plasticizer improves the two polymers’ ability to mix together at the interface [34]. The seaweed’s mechanical qualities are improved by the potent hydroxyl group interactions between the cinnamon nanoparticles and the seaweed [35].

The greater agglomeration point was likely the cause of the drop in TS following the optimal loading. The tension created by the aggregation point reduced the intermolecular contact between the macroalgae matrix and nanofillers. The composite films’ flexibility is indicated by the elongation result. The versatility of elastic and flexible plastic products is crucial in industries including cosmetics, agriculture, and food packaging [36]. The control film did not exhibit as much elongation as the 1% films did. The loading of nano cinnamon from 1% to 5% gradually causes the biocomposite’s elongation to rise. The film’s elongation, however, dropped when 7% nanofiller was added. The macroalgae–glycerol (plasticizer) connection may be disrupted by the addition of cinnamon nanoparticles at high concentrations (7%), leading to a reduction in the elasticity and flexibility of the composite films. Overall, it is evident that the mechanical properties of the films were enhanced by the addition of cinnamon nanoparticles [37].

The SEM images displayed in Figure 5b present the surface morphology of fractured seaweed-based films incorporated with cinnamon nanoparticles (3% and 5%) based on the highest and lowest tensile strength values obtained from mechanical tests and control sample film for differentiation at 100× and 500× magnifications. When compared to the fracture surface of the control film at 100× magnification, the shattered surfaces of the seaweed-based films containing 3 and 5% filler displayed a more compact shape with fewer pores. This indicates the strong interaction between the seaweed and filler, which may have resulted in good interfacial stress transfer [33]. This is also reinforced by mechanical and physical tests, which showed that the 5% cinnamon nanoparticle concentration improved the physical and mechanical properties of the seaweed film, demonstrating that the fillers were evenly distributed throughout the matrix. Some holes can be seen in the morphology of the control seaweed-based films, which suggests that they have worse mechanical capabilities than the seaweed-based films that incorporate cinnamon nanoparticles.

**Figure 6:**
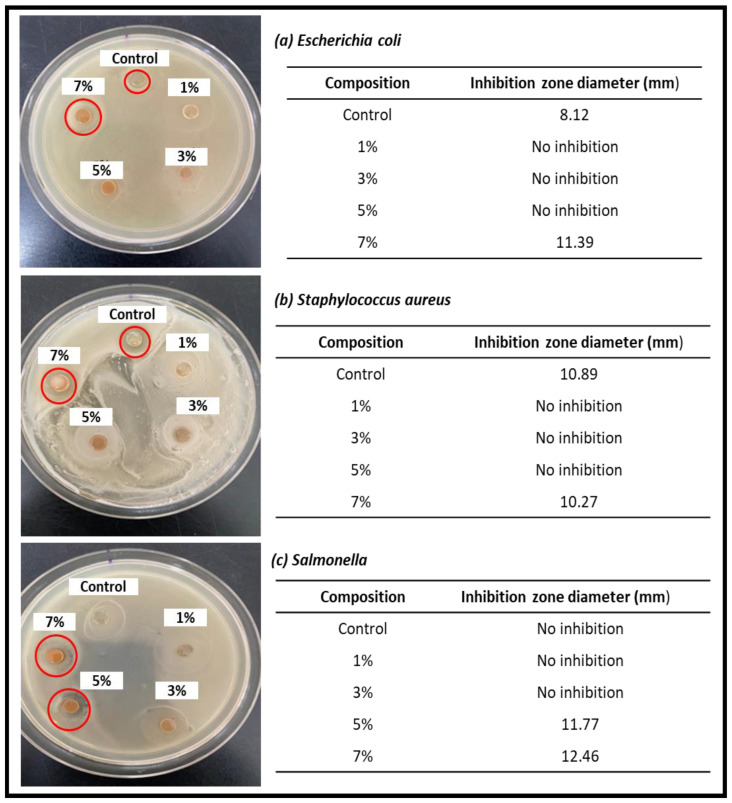
Antimicrobial properties of the seaweed-based cinnamon film against (**a**) *E. coli*, (**b**) *S. aures*, and (**c**) *Salmonella*.

Wide-ranging Gram-negative and Gram-positive bacteria are inhibited by cinnamon. Consequently, this study was performed to identify cinnamon’s potential to inhibit the growth of bacteria. Figure 6 shows the antimicrobial test results of seaweed-based films incorporated with cinnamon nanoparticles against each tested bacterium: *Escherichia coli* (*E. coli*), *Staphylococcus aureus* (*S. aureus*), and Salmonella. The results of the antimicrobial test for each bacterium with different samples of seaweed films incorporated with cinnamon nanoparticles are displayed in Figure 6. The diameter of the inhibition zone for each sample was recorded by using a digital caliper. The seaweed film incorporated with 7% cinnamon nanoparticles has antimicrobial activity against each of the tested bacteria. Cinnamon will definitely have antimicrobial effects on other prevalent foodborne bacteria, such as Salmonella and Campylobacter, if it can eliminate *E. coli*, one of the most virulent foodborne germs known to exist today [19]. An unrecordable inhibition zone was not valid. The growth of test bacteria showed isolated colonies or less than semi-confluent growth. The zone showed distortion from a circular shape and was not within tolerance limits set. The addition of cinnamon to the matrix structure significantly improved the mechanism of the film’s antibacterial activity, which has noticeable effects on the surface structure and functional properties of the biopolymer film [38]. Although the mechanical properties of 7% cinnamon nanoparticles were not the best, it is still applicable for food packaging applications. Our findings indicate the high potential of the antibacterial film in several food and non-food packaging applications.

## 4. Conclusions

A seaweed-based bio-nanocomposite film incorporated with cinnamon nanoparticles was successfully prepared and characterized. The results revealed that the significant action of the biopolymer matrix was remarkably enhanced by the incorporation of cinnamon particles in the film’s components, as demonstrated by the morphology of the film. The 5% concentration of cinnamon nanoparticles led to the highest strength compared to other concentrations. The introduction of the 1% nano cinnamon concentration already showed positive results for the strength compared to the control film without the filler. This indicates that the addition of cinnamon nanoparticles enhanced the strength of the biopolymer film. The hydrophobicity of the film gradually increased as the water contact angle values increased due to the insertion of cinnamon nanoparticles and their interaction in the film structure. Furthermore, the addition of cinnamon to the film caused a slight increase in the degradation temperature, indicating the thermal stability of the film, with the increase in cinnamon nanoparticles in the films. The presence of strong hydrogen bonds is suggested by FT-IR results, indicating that strong hydrogen bonding influences the properties of the biopolymer film. The antimicrobial properties of cinnamon nanoparticles are shown through the effective outcome against foodborne bacteria. The optimal inhibition zone diameters against *E. coli*, *S. aureus*, and *Salmonella* were 11.39 mm, 10.27 mm, and 12.46 mm, respectively, with the incorporation of 7% cinnamon. This shows that the addition of cinnamon nanoparticles to the biopolymer film creates antimicrobial properties. Although the mechanical properties of 7% cinnamon nanoparticles were not optimal, they are still in average ranges and applicable for food packaging applications. The results of the functional properties indicated that the seaweed-based 7% cinnamon-nanoparticle-reinforced film can potentially be utilized for packaging applications.

## Data Availability

Not applicable.

## References

[1] Rodrigues M., Abrantes N., Gonçalves F., Nogueira H., Marques J., Gonçalves A. (2019). Impacts of plastic products used in daily life on the environment and human health: What is known?. Environ. Toxicol. Pharmacol..

[2] Xanthos D., Walker T.R. (2017). International policies to reduce plastic marine pollution from single-use plastics (plastic bags and microbeads): A review. Mar. Pollut. Bull..

[3] Chatterjee S., Sharma S. (2019). Microplastics in our oceans and marine health. Field Actions Sci. Rep. J. Field Actions..

[4] Zaki M., Atiqah M.N., Abdul Khalil H.P.S., Ikram H., Alfatah T., Mistar E., Adisalamun A., Yahya E.B. (2022). Microbial enhancement of nanocellulose isolation from sawn timber industrial wastes and fabrication of biocomposite membranes. Bioresour. Technol. Rep..

[5] Onen Cinar S., Chong Z.K., Kucuker M.A., Wieczorek N., Cengiz U., Kuchta K. (2020). Bioplastic production from microalgae: A review. Int. J. Environ. Res. Public Health.

[6] Abdul Khalil H.P.S., Yahya E.B., Jummaat F., Adnan A., Olaiya N., Rizal S., Abdullah C., Pasquini D., Thomas S. (2022). Biopolymers based aerogels: A review on revolutionary solutions for smart therapeutics delivery. Prog. Mater. Sci..

[7] Moshood T.D., Nawanir G., Mahmud F., Mohamad F., Ahmad M.H., AbdulGhani A. (2022). Biodegradable plastic applications towards sustainability: A recent innovations in the green product. Clean. Eng. Technol..

[8] Jiang T., Duan Q., Zhu J., Liu H., Yu L. (2020). Starch-based biodegradable materials: Challenges and opportunities. Adv. Ind. Eng. Polym. Res..

[9] Muhammad S., Abdul Khalil H.P.S., Abd Hamid S., Albadn Y.M., Suriani A., Kamaruzzaman S., Mohamed A., Allaq A.A., Yahya E.B. (2022). Insights into Agricultural-Waste-Based Nano-Activated Carbon Fabrication and Modifications for Wastewater Treatment Application. Agriculture.

[10] Lorenzo J.M., Munekata P.E., Dominguez R., Pateiro M., Saraiva J.A., Franco D. (2018). Main groups of microorganisms of relevance for food safety and stability: General aspects and overall description. Innovative Technologies for Food Preservation.

[11] Atiwesh G., Mikhael A., Parrish C.C., Banoub J., Le T.-A.T. (2021). Environmental impact of bioplastic use: A review. Heliyon.

[12] Lomartire S., Marques J.C., Gonçalves A.M. (2022). An Overview of the Alternative Use of Seaweeds to Produce Safe and Sustainable Bio-Packaging. Appl. Sci..

[13] Buschmann A.H., Camus C., Infante J., Neori A., Israel Á., Hernández-González M.C., Pereda S.V., Gomez-Pinchetti J.L., Golberg A., Tadmor-Shalev N. (2017). Seaweed production: Overview of the global state of exploitation, farming and emerging research activity. Eur. J. Phycol..

[14] Emadian S.M., Onay T.T., Demirel B. (2017). Biodegradation of bioplastics in natural environments. Waste Manag..

[15] Wu J., Sun X., Guo X., Ge S., Zhang Q. (2017). Physicochemical properties, antimicrobial activity and oil release of fish gelatin films incorporated with cinnamon essential oil. Aquac. Fish..

[16] Yahya E.B., Abdul Khalil H.P.S., Ahmad M.I., Rizal S., Muhammad S. (2023). Cleaner approach of preparing antibacterial bioaerogel scaffolds using oil palm waste nanocellulose. Ind. Crops Prod..

[17] Dhillon G., Amarjeet K. (2013). Quality evaluation of bread incorporated with different levels cinnamon powder. Int. J. Food Sci. Nutr. Diet.

[18] Cox S.D., Markham J. (2007). Susceptibility and intrinsic tolerance of *Pseudomonas aeruginosa* to selected plant volatile compounds. J. Appl. Microbiol..

[19] Sun D., Lv J., Chen W., Li S., Guo Y., Bian Z., Yu C., Zhou H., Tan Y., Chen J. (2014). Spicy food consumption is associated with adiposity measures among half a million Chinese people: The China Kadoorie Biobank study. BMC Public Health.

[20] Frank K., Garcia C.V., Shin G.H., Kim J.T. (2018). Alginate biocomposite films incorporated with cinnamon essential oil nanoemulsions: Physical, mechanical, and antibacterial properties. Int. J. Polym. Sci..

[21] Oyekanmi A., Abdul Khalil H.P.S., Rahman A., Mistar E., Olaiya N., Alfatah T., Yahya E.B., Mariana M., Hazwan C., Abdullah C. (2021). Extracted supercritical CO_2_ cinnamon oil functional properties enhancement in cellulose nanofibre reinforced Euchema cottoni biopolymer films. J. Mater. Res. Technol..

[22] Salim A.A., Bidin N., Ghoshal S.K. (2018). Growth and characterization of spherical cinnamon nanoparticles: Evaluation of antibacterial efficacy. LWT.

[23] Lixourgioti P., Goggin K.A., Zhao X., Murphy D.J., van Ruth S., Koidis A. (2022). Authentication of cinnamon spice samples using FT-IR spectroscopy and chemometric classification. LWT.

[24] Li Y.-q., Kong D.-x., Wu H. (2013). Analysis and evaluation of essential oil components of cinnamon barks using GC–MS and FTIR spectroscopy. Ind. Crops Prod..

[25] Lin R.-C., Mohamed M.G., Chen T., Kuo S.-W. (2017). Coumarin-and carboxyl-functionalized supramolecular polybenzoxazines form miscible blends with polyvinylpyrrolidone. Polymers.

[26] Huang Z., Jia S., Zhang L., Liu X., Luo Y. (2019). Inhibitory effects and membrane damage caused to fish spoilage bacteria by cinnamon bark (*Cinnamomum tamala*) oil. LWT.

[27] Abhay A.K., Rakesh S.K., Nishant K., Birendra P. (2021). Preparation of superfine cinnamon bark nanocrystalline powder using high energy ball mill and estimation of structural and antioxidant properties. IOP Conf. Ser. Mater. Sci. Eng..

[28] Tye Y.Y., HPS A.K., Kok C.Y., Saurabh C.K. (2018). Preparation and characterization of modified and unmodified carrageenan based films. IOP Conf. Ser. Mater. Sci. Eng..

[29] Stupnik M., Kolosov V., Kalinichenko V., Pismennyi S. (2014). Physical modeling of waste inclusions stability during mining of complex structured deposits. Progress. Technol. Coal Coalbed Methane Ores Min..

[30] Chen P., Tian H., Zhang L., Chang P.R. (2008). Structure and properties of soy protein plastics with ε-caprolactone/glycerol as binary plasticizers. Ind. Eng. Chem. Res..

[31] Zhou Y., Wu X., Chen J., He J. (2021). Effects of cinnamon essential oil on the physical, mechanical, structural and thermal properties of cassava starch-based edible films. Int. J. Biol. Macromol..

[32] Ramadhani F.S., Rostini I., Anna Z., Rochima E. (2019). Characterization of edible film from seaweed flour (*Eucheuma cottonii* Weber-van Bosse, 1913) with different types of plasticizer. World Sci. News.

[33] Chong E., Jafarzadeh S., Paridah M., Gopakumar D.A., Tajarudin H., Thomas S., Abdul Khalil H. (2019). Enhancement in the physico-mechanical functions of seaweed biopolymer film via embedding fillers for plasticulture application—A comparison with conventional biodegradable mulch film. Polymers.

[34] Surya I., Olaiya N., Rizal S., Zein I., Sri Aprilia N., Hasan M., Yahya E.B., Sadasivuni K.K., Abdul Khalil H.P.S. (2020). Plasticizer enhancement on the miscibility and thermomechanical properties of polylactic acid-chitin-starch composites. Polymers.

[35] Zhang X.-y., Chen Y.-p., Han J., Mo J., Dong P.-f., Zhuo Y.-h., Feng Y. (2019). Biocompatiable silk fibroin/carboxymethyl chitosan/strontium substituted hydroxyapatite/cellulose nanocrystal composite scaffolds for bone tissue engineering. Int. J. Biol. Macromol..

[36] Montes M.I., Cyras V.P., Manfredi L.B., Pettarin V., Fasce L.A. (2020). Fracture evaluation of plasticized polylactic acid/poly (3-HYDROXYBUTYRATE) blends for commodities replacement in packaging applications. Polym. Test..

[37] Hosseini M., Razavi S., Mousavi M. (2009). Antimicrobial, physical and mechanical properties of chitosan--based films incorporated with thyme, clove and cinnamon essential oils. J. Food Process. Preserv..

[38] Xu T., Gao C., Yang Y., Shen X., Huang M., Liu S., Tang X. (2018). Retention and release properties of cinnamon essential oil in antimicrobial films based on chitosan and gum arabic. Food Hydrocoll..

